# Correlates between Feeding Ecology and Mercury Levels in Historical and Modern Arctic Foxes (*Vulpes lagopus*)

**DOI:** 10.1371/journal.pone.0060879

**Published:** 2013-05-06

**Authors:** Natalia Bocharova, Gabriele Treu, Gábor Árpád Czirják, Oliver Krone, Volker Stefanski, Gudrun Wibbelt, Ester Rut Unnsteinsdóttir, Páll Hersteinsson, Gereon Schares, Lilia Doronina, Mikhail Goltsman, Alex D. Greenwood

**Affiliations:** 1 Department of Vertebrate Zoology, Faculty of Biology, M.V. Lomonosov Moscow State University, Moscow, Russia; 2 Department of Wildlife Diseases, Leibniz Institute for Zoo and Wildlife Research, Berlin, Germany; 3 Behaviorial Physiology of Farm Animals, University of Hohenheim, Stuttgart, Germany; 4 Department of Life and Environmental Science, University of Iceland, Reykjavik, Iceland; 5 Institute for Epidemiology, Friedrich-Loeffler-Institut, Federal Research Institute for Animal Health, Wusterhausen, Germany; Federal University of Rio de Janeiro, Brazil

## Abstract

Changes in concentration of pollutants and pathogen distribution can vary among ecotypes (e.g. marine versus terrestrial food resources). This may have important implications for the animals that reside within them. We examined 1) canid pathogen presence in an endangered arctic fox (*Vulpes lagopus*) population and 2) relative total mercury (THg) level as a function of ecotype (‘coastal’ or ‘inland’) for arctic foxes to test whether the presence of pathogens or heavy metal concentration correlate with population health. The Bering Sea populations on Bering and Mednyi Islands were compared to Icelandic arctic fox populations with respect to inland and coastal ecotypes. Serological and DNA based pathogen screening techniques were used to examine arctic foxes for pathogens. THg was measured by atomic absorption spectrometry from hair samples of historical and modern collected arctic foxes and samples from their prey species (hair and internal organs). Presence of pathogens did not correlate with population decline from Mednyi Island. However, THg concentration correlated strongly with ecotype and was reflected in the THg concentrations detected in available food sources in each ecotype. The highest concentration of THg was found in ecotypes where foxes depended on marine vertebrates for food. Exclusively inland ecotypes had low THg concentrations. The results suggest that absolute exposure to heavy metals may be less important than the feeding ecology and feeding opportunities of top predators such as arctic foxes which may in turn influence population health and stability. A higher risk to wildlife of heavy metal exposure correlates with feeding strategies that rely primarily on a marine based diet.

## Introduction

Ecotype differences (e.g. food resources, predator-prey or host-pathogen interactions) can have profound impacts on organismal adaptation and long term survival. For example, island populations are particularly vulnerable to decline or extinction [1]. Extended isolation in an ecosystem with relatively poor biodiversity and a stable environment can lead to habitat specialization, loss of genetic diversity and thus the reduced ability to adjust if the environment suddenly changes. Part of that environment includes pathogens and pollutants which in the case of pathogens can suddenly emerge and in the case of pollutants increase in concentration rapidly and disperse throughout the food chain. In addition to their clinical effects, pollutants can cause immune suppression which can exacerbate the effect of infection even for normally low or non-pathogenic microorganisms. These effects can be further aggravated by climate change causing energetic stress associated with low food accessibility and starvation, resulting in population decline. Thus, determining the distribution of pathogens and pollutants among ecosystems are critical to identifying threats to population and species health.

Arctic foxes (*Vulpes lagopus*, formerly known as *Alopex lagopus*) vary dramatically in terms of threats to population sustainability. On the Russian Commander Islands (Mednyi and Bering Islands) in the Bering Sea the arctic fox populations have been isolated since the Pleistocene [2] and are thought to represent two endemic subspecies [3]. Until the mid-20^th^ century, the population on Mednyi Island remained stable at about 1000 foxes [4] but crashed between 1970–1980 [5]. During the crash period the foxes exhibited different clinical signs such as mange, low body weight, and very high cub mortality (88–96.4%) that have resulted in a continuous decrease in population size [6]. Currently the population consists of ca. 100 individuals [7]. In contrast, the Bering Island population has not suffered serious declines. However, the two Commander Island populations are somewhat different as the Bering Island is larger, human inhabited and thus, a greater variety of food sources, such as rodents and waste, are available [8].

Islands are not the only ecotype inhabited by arctic foxes. They represent a circumpolar species and have a wide distribution in Iceland, Scandinavia, the Russian Federation, Greenland, Alaska and Canada [9]. Traditionally arctic foxes are separated into two ecotypes – ‘inland’ (or ‘lemming’) and ‘coastal’ [10]. Arctic foxes are top-predators in their environment and scavengers that generally rely on small mammals such as lemmings and voles. When rodent prey are rare or absent they feed on polar bear leftovers, on carcasses of different marine vertebrate species, birds and eggs, fish and marine invertebrates [11]. Thus, across ecotypes, they may be exposed to different pathogens and pollutants or different levels of both. However, in general, the island populations are at greater risk of decline than non-island populations as a consequence of their isolation [12], [13].

High concentrations of heavy metals and organochlorines were reported for Arctic fauna in the last decades [14], [15]. Such pollutants are known to accumulate in the higher levels of the food chain, suppress the immune system [16] and negatively affect reproduction [17]. Mercury (Hg) presents health risks to arctic wildlife and human populations as it can reach concentration levels of toxicological significance, especially in high trophic level marine species like seabirds, seals and polar bears [17]–[19]. During the last three decades an increased long-range transport and deposition of atmospheric Hg of anthropogenic origin has been documented in the Arctic [20]. An estimated 200 t of Hg are transported each year from the mid-latitudes into the Arctic from anthropogenic and natural sources by atmospheric processes, ocean currents, coastal erosion and rivers [21]. This trend will likely continue as models indicate that there will be a 25% increase in Hg emissions from human sources worldwide in 2020 compared to 2005 [22]. Once Hg is present in polar regions, it accumulates due to surface deposition and it is released again during snow melt entering the marine food chain where it is metabolized to a more toxic organic Hg form through microbial methylation [23]. As Hg (both organic and inorganic) biomagnifies in food chains and bioaccumulates in tissues, foxes feeding mainly on marine prey suffer high Hg burdens.

To determine the ecotype profile for Hg exposure of arctic foxes and attempt to explain the unusual arctic fox population decline in Mednyi Island, we screened for multiple plausible pathogens that could cause mass mortality in canids taking into consideration, the island has not been inhabited since the 1960's and the only food sources for the foxes are sea birds and seals [8]. In addition, comparative THg levels were measured in hair from historical specimens from the Commander Islands that correlated with the population pre-crash period on Mednyi Island, modern Mednyi foxes and two unrelated and geographically remote ecotypes represented by inland and coastal Icelandic arctic fox populations. Where possible, food THg levels were measured as well. The results do not suggest a pathogen cause for the decline on Mednyi Island. However, ecotype and age strongly predicts THg levels in arctic foxes. The results are discussed with respect to the peculiar decline of arctic foxes on Mednyi Island and Hg exposure risk associated with terrestrial ecotypes dependent on resources derived from marine environments.

## Methods

### Study area

**Figure 1:**
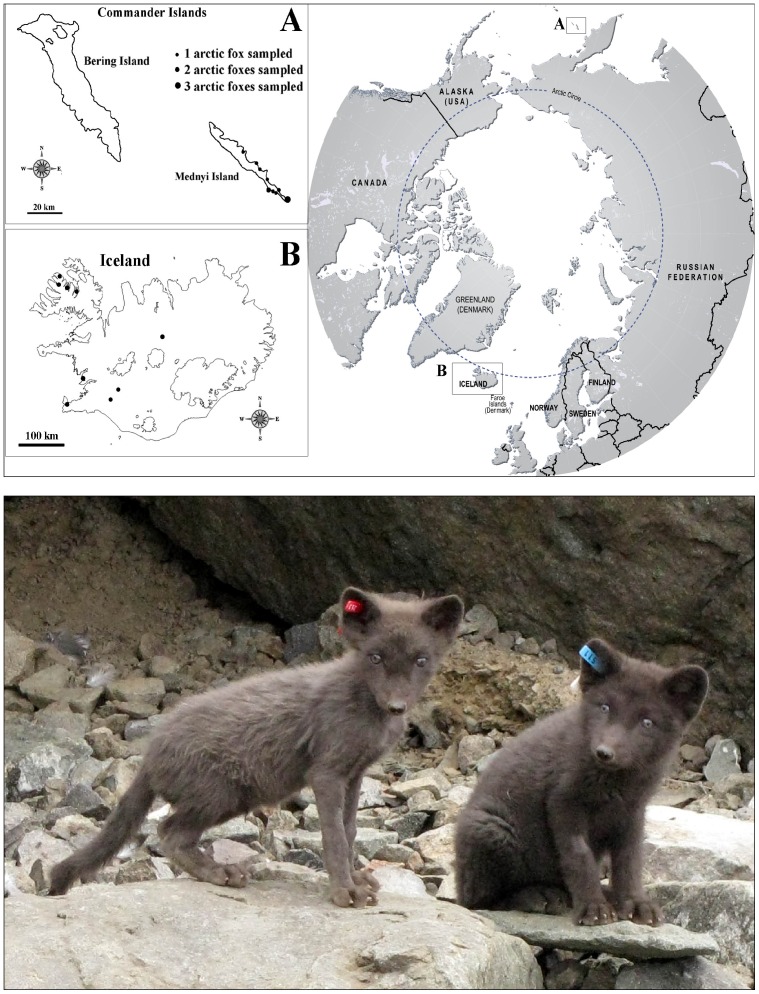
Sampling localities and health condition of Mednyi Island arctic foxes. Localities from which samples were derived are shown by black dots in panel A. The number of samples taken at each Mednyi Island location site vary by dot size as indicated. A refers to the Commander Island and B Iceland which are then shown relative to their circumpolar location. The historical samples cannot be assigned to a precise locality within a geographic region Commander Islands. Panel B shows an image representing two Mednyi Island foxes demonstrating different health status. The fox on the left (red ear tag) is underweight with poor coat condition and the fox on the right (blue ear tag) presents good general health condition.

The Commander Islands are situated in the northern Pacific Ocean, to the West from Aleutian Island arc and 175 km to the East from Kamchatka peninsula between 55°25′ and 54°31′N and 165°04′ and 168°E (Figure 1A). The archipelago consists of 15 islands of different sizes, the two biggest are the Bering Island and the Mednyi Island divided by a 49 km wide strait. The remaining 13 islands do not exceed 1.5 km in diameter and have no terrestrial fauna. The Bering Island is 90 km long and 5–40 km wide, 1667 km^2^. The Mednyi Island is smaller, 53 km long and 0.35–7.5 km wide, and covers an area of 186 km^2^. Both islands are the upper part of an underwater mountain chain that goes from the South-East to North-West, with a maximum altitude of 755 m on the Bering Island and 647 m on the Mednyi Island.

The mountains are tundra habitat. Foxes are restricted to the narrow coastline where their bird colonies and seals rookeries are concentrated. The main prey species on the Mednyi Island are northern fulmar (*Fulmarus glacialis rodgersii*) and storm petrels (*Oceanodroma furcata* and *O. leucorhoa*), followed by alcids (*Alcidae*), cormorants (*Phalacrocorax pelagicus* and *Ph. urile*), marine invertebrates and otarid rookery products [7]. The diet of the Bering arctic fox also consists of marine birds and seals rookery scavenging. Two terrestrial mammals were introduced to the Bering Island at the end of the 19^th^ century: the red vole (*Clethrionomys rutilus*) was introduced accidentally in 1870 and reindeer (*Rangifer tarandus*) were introduced on purpose in 1882 [24]. Both serve as alternative food resource for the Bering Island arctic foxes [8].

The arctic fox is the only native terrestrial mammal in Iceland. The species is found in most regions but the density varies and is probably highest in the Westfjords (Hersteinsson, *unpubl. data*) where there is proportionally long and productive coast line compared to land area and some of the largest bird cliffs in the country. The Icelandic population comprises both “coastal” and “inland” fox ecotypes, with regard to the main food resources [25]. Wood mice (*Apodemus sylvaticus*) are common in all vegetated areas [26]. The coastal ecotype foxes mainly feed on sea birds and eggs, invertebrates and sea mammal carcasses. Inland foxes feed on ptarmigans, migrating waders, geese, eggs and carrion [25].

### Collection of modern and historical arctic fox and prey samples

As a part of annual summer counts on Mednyi Island, cubs and adults were caught with live traps [0.4×0.4×1 m] or by hand for ear tagging and weight measurement. About 70% of animals in the study area have individual tags and known life history. Animals were marked with plastic ear tags sized 3.5×1 cm (D 400 Rototag-Ohrmarken, Fa. Horn, Horn-Tierzuchtgeräte, Dülmen, Germany). The tags were placed in the cartilagous part of the pinna of animals using Rototag-forceps (D 410, Fa. Horn) after disinfecting the ears (Figure 1B). The entire handling process took <10 min.

For pathogen screening of Mednyi Island foxes, 72 blood samples (21 adults and 51 juveniles), 18 skin samples (14 adults and 4 juveniles) and 18 faecal samples (10 adults and 8 juveniles) were taken during the field seasons 2006 and 2008. The blood samples were taken by the venipuncture of the jugular vein. The blood was collected in sterile tubes and centrifuged after 1 to 4 hours at 3000 rpm for 10 minutes. The serum was separated, aliquoted and frozen in liquid nitrogen for 1–2 months (in the field). Sera were subsequently kept in the laboratory at −20°C (2006) or −80°C (2008). After centrifugation, the remaining blood clot was fixed with 70% ethanol and stored at room temperature (for 1–2 months in the field) and −20°C in the laboratory. Skin samples of 4 to 6 mm^2^ were cut from the ear edge with a surgical scissors and fixed with RNA-later® (Quiagen). The wound was treated with Terramycin spray (Pfizer Limited). Skin samples were kept in liquid nitrogen for 1–2 months in the field and at −80°C in the laboratory until analysis. Faeces samples were taken if they could be associated with a specific fox, frozen in liquid nitrogen in the field and then kept at −80°C.

For THg measurements, 12 fur samples (6 adults and 6 juveniles) were taken on Mednyi Island during 2011 field season. Additionally, bird and seal organs and fur were collected to estimate THg levels in fox prey species. The prey species tissues were sampled from carcasses in the fur seals rookeries or food remains on arctic fox den sites on the Mednyi Island during 2010 and 2011 field seasons. About 1 g of each tissue was air-dried and kept at room temperature until analysis. Approximately 1 g of fur was collected from 2–3 cm of square of skin with surgical scissors. Hairs were cut close to the skin and kept in a sterile plastic bag at room temperature.

For ecotype comparison, 28 hair samples (16 adults, 11 juveniles, 1 unknown) from arctic foxes from Iceland collected during the 2011–2012 hunting season were used. Fur was cut from neck, sides or hind legs with small scissors. Age of the Iceland arctic foxes was determined by X-ray measurement and pulp cavity volume determination [27].

Historical samples were taken from the pelts of arctic foxes (*n* = 11) and northern fur seals (*Callorhinus ursinus*) (*n* = 17) from the collections of the Zoological Museum of the Zoological Institute of Russian Academy of Sciences (Saint Petersburg) and the Zoological Museum of Moscow State University. The pelts were collected from 1910 to 1984 on Commander Islands. Where possible, equal sex ratios between the different ages or populations was maintained for sampling (see Tables S1 and S2).

### Pathogen screening

Serum samples from adult and juvenile Mednyi Island foxes were tested for specific antibodies against *Toxoplasma gondii*, *Neospora caninum*, Canine Parvovirus and Canine Distemper virus (Table 1). Thirty one serum samples from the 2006 field season (6 adults and 25 cubs) and 27 serum samples from the 2008 field season (12 adults and 15 cubs) were used for screening for antibodies against the two protozoa which might cause prenatal infection via transplacental transmission and thus involved in abortion or disease in congenitally infected cubs (*Toxoplasma gondii* and *Neospora caninum*). The specific antibodies were tested by commercial enzyme-linked immunosorbent assays (ELISA) (in case of Toxoplasma) and immunoblotting for confirmation. For *N. caninum* only immunoblotting was used. The confirmatory analysis for *Toxoplasma* and tests for *Neospora* were done by the German National Reference Laboratory for Toxoplasmosis (Friedrich-Loeffler-Institute, Wusterhausen, Germany).

**Table 1:** Results of pathogen screening in Mednyi Island population.

Pathogen	Test detects antigen/antibody (AG/Ab), tissue type	Number of samples (adults+juveniles)	Number of positive samples/percentage of positive foxes
*Brucella* spp.	AG, blood	13 adults	0
Herpesvirus	AG, skin	13 adults+5 juveniles	0
Canine Parvovirus	AG, feces	10 adults+4 juveniles	0
	Ab, sera	7 adults+30 juveniles	0
Morbilliviruses	AG, blood	13 adults+10 juveniles	0
	Ab, sera	6 adults+30 juveniles	0
Caliciviruses	AG, blood, feces	13 adults+10 juveniles,10 adults+4 juveniles	0
Reovirurses	AG, blood, feces	13 adults+10 juveniles,10 adults+4 juveniles	0
*Neospora caninum*	Ab, sera	18 adults+40 juveniles	0
*Toxoplasma gondii*	Ab, sera	18 adults+40 juveniles	3 adults/5%

Tests used, number of samples and test results are indicated.

Thirty seven serum samples (7 adults and 30 juveniles) were tested for Canine Parvovirus by haemagglutination assays and 36 serum samples (6 adults and 30 juveniles) for Canine Distemper virus by direct neutralizing peroxidase-linked antibody (NPLA) assay. Details of the serological methods can be found in Text S1.

DNA was extracted from blood and skin samples with DNeasy Blood and Tissue kit (QIAGEN) according to manufacturer instruction. The blood clots were processed as tissue samples. RNA was extracted from blood clots with RNeasy Mini kit (QIAGEN). For nucleic acid extraction from feces the RTP DNA/RNA Virus Mini kit (Invitek) was used according to the manufacturer's instruction. RNA and DNA concentrations were determined spectrophotometrically.

DNA extracted from blood clots (13 adults), skin (13 adults and 5 juveniles with skin lesions) and faeces (10 adults and 4 juveniles) was used for PCR for *Brucella spp*, Canine Herpesvirus and Parvovirus screening respectively (Table 1). Coloreless GoTaq® Reaction buffer (pH 8.5, 7.5 mM MgCl2) and GoTaq® DNA Polymerase (both Promega, Germany) were used for the PCR. The conditions are described in the Table S3 and the primers in Table S4. PCR products were analyzed by agarose gel electrophoresis followed by visualization under UV light.

RNA samples extracted from blood clots (13 adults and 10 juveniles) were used for Morbillivirus, Calicivirus and Reovirus reverse transcription PCR with SuperScript RT-PCR Platinum Taq® one step (Invitrogen) (Table 1). For Calicivirus and Reovirus we also tested RNA extracted from faecal samples (10 adults and 4 juveniles) using the same primers and reagents as for the blood clots (Text S1). Details of the reverse transcription and qPCR analysis are described in Text S1.

### THg determination from hair and tissue samples

There was no information available about which chemicals have been used to preserve the museum specimens we collected the hair samples from. Formerly mercuric compounds were commonly used as a preservative in taxidermy [28]. Thus, we fully removed external mercury contamination by washing all hair samples five times with ultra-clean de-ionized water (Merck Millipore, Germany) with 1% HCl [29]. Each sample was put in a glass vial and shaken in the cleaning solution for at least 5 minutes. After shaking out the excess rinsate, the vessels were placed in an oven at 50°C over night to allow the hair to dry. Microwave assisted acid digestion using a micro PREP 1500 (MLS, Germany) was carried out to obtain THg. About 0.3 g of hair or dried tissue samples were directly weighed in PTFE vials. One ml concentrated HCl, 6 ml of HNO_3_ (37%) and 1 ml of H_2_O_2_ (30%) were added to each flask and microwaved for 1 hour 4 steps up to 200°C. Following digestion, samples were transferred to 25 ml volumetric flasks and filled up with ultrapure water. The method was validated on certified reference material (Tuna Muscle, ERM-CE464) which was also microwave digested.

THg levels were determined with a ZEENIT 700 absorption spectrometer (Analytik Jena, Germany) with Hg-hydride system using the cold-vapor technique. Sodium borohydride was used as a reducing agent and argon gas as a carrier. The detection limit was 0.07 µg/L based on 5 ml sample volume. Calibration was done on a 5-point curve using the mean of 3 repeated measure points. All THg concentrations (mg/kg per sample) were calculated as dry weight (d.w.) and blank corrected by the AAS (WinAAS 3.22.0).

### Statistical analysis

Analyses were performed using JMP 5.0.1, the level of significance being set to α = 0.05, while 0.05<*P*<0.1 was considered as a trend. THg concentration in arctic fox hair samples was not normally distributed. Thus, we normalized the data by root square transformation and these values were used in all subsequent analyses. The THg level was not significantly different between months (*F*_9,39_ = 0.578, *P* = 0.80), so we pooled the data.

The effect of ecotype, age, sex and their biologically relevant interaction on THg concentration in hair was analysed using a general linear model (GLM). The ecotype of the samples was defined as “*Commander Islands* – *museum*”, “*Mednyi Island* – *modern*”, “*Iceland* – *coastal*” and “*Iceland* – *inland*”, thus including both the geographical and sampling (museum vs. modern) origin and the feeding strategy (coastal vs. inland). Tukey-Kramer post-hoc test was used to determine differences in THg levels between different populations with different ecotypes.

Since age had a significant effect on THg levels from arctic fox hair (see results and discussion), after excluding all the individuals with unknown age, we analyzed the effect of ecotype and sex in the two age groups separately (adults *n* = 27, juveniles *n* = 20). In the case of juveniles, we could not normalize the data and additionally, the sample size for the different ecotype groups was low. Thus, non-parametric tests were employed.

## Results

### Pathogen screening

All tested foxes were found to be seronegative for Canine Parvovirus, Canine Distemper and *Neospora caninum* (see Table 1). Three adult foxes were seropositive for *Toxoplasma gondii* (17% of tested adults, 5% of all tested foxes). Both ELISA and immunoblotting yielded the same results. Quantitative PCR analysis of 18S rRNA suggested the samples were of sufficient quality for detection of pathogens with RNA based genomes. However, PCRs for all tested DNA and RNA genome pathogens were negative (see Table 1).

### THg levels in arctic foxes

**Figure 2:**
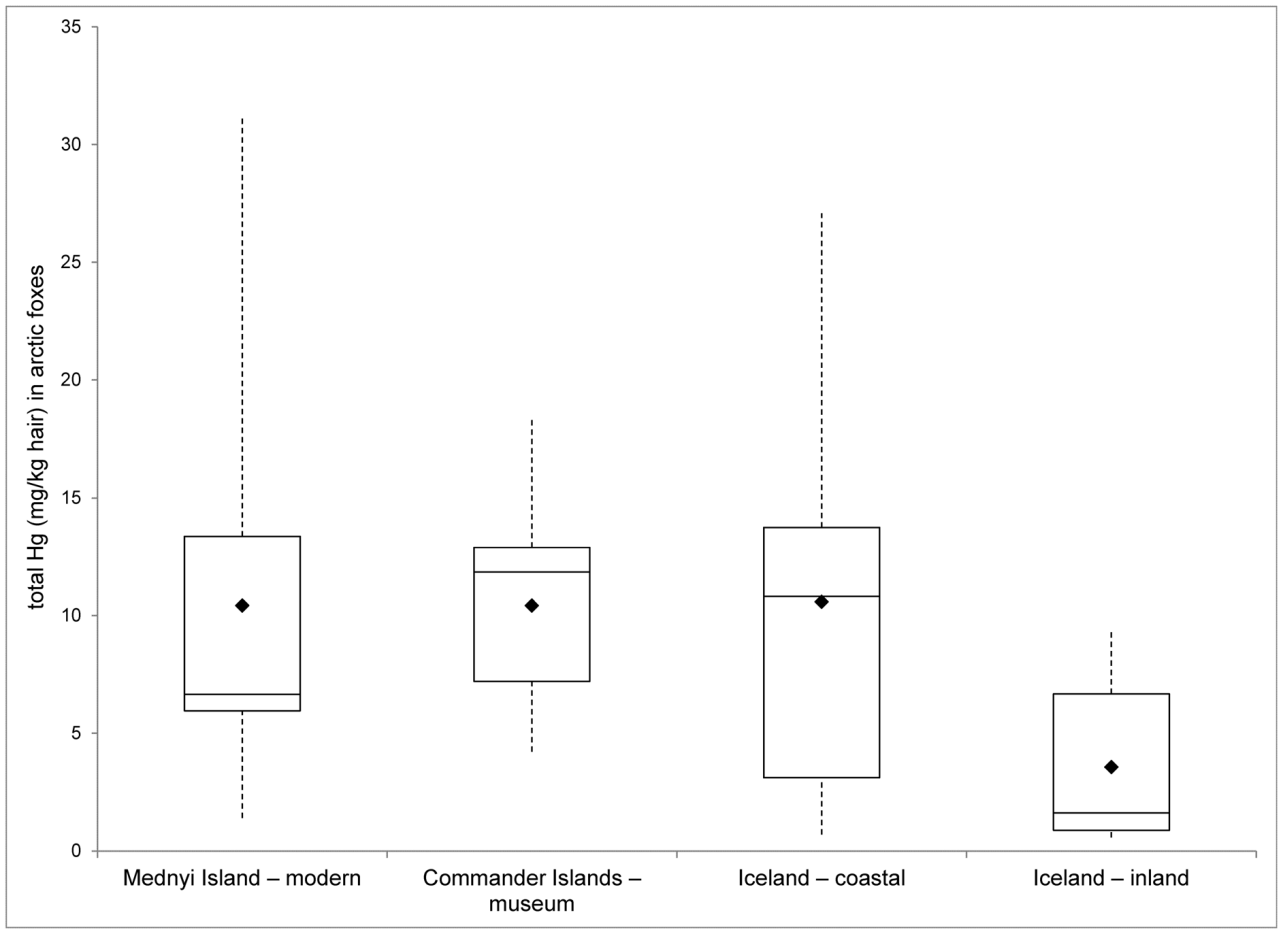
Levels of THg (mg/kg d.w.) in hair of arctic foxes originating from different ecotypes. Box plots indicate median (solid line) and mean (black diamond) of the THg values. The ranges of values are shown as dashed lines.

Concentrations of THg found in arctic fox hair in the four ecotypes are presented in Table 2. Arctic foxes of different ecotype had significantly different THg concentration in their hair (*F*_3,35_ = 4.479, *P* = 0.009). There was no significant difference between the average levels of the THg in arctic foxes originating from the Commander Islands (both museum and modern) and Iceland coastal populations (Tukey-Kramer post-hoc test, all *P*>0.05), while those from the Iceland inland ecotype had a significantly lower average heavy metal concentration compared to the other three populations (*P*<0.05) (Table 2, Figure 2).

**Table 2:** Summary statistics of the concentration (mg/kg d.w.) of total mercury in hair of arctic foxes from the Commander Islands and Iceland by ecotype and age.

	Total Hg (mg/kg d.w. hair)	Commander Islands – museum	Mednyi Island – modern	Iceland – coastal	Iceland – inland
Both age groups	Mean ± SE	10.42±1.31	10.42±2.45	10.58±2.12	3.55±1.00
	range	4.21–18.34	1.39–31.17	0.699–27.08	0.57–9.28
	*n*	11	12	16	12
Adults	Mean ± SE	9.58±2.72	15.50±3.80	14.52±2.51	2.89±1.31
	range	4.21–18.34	6.59–31.17	2.36–27.08	0.57–7.68
	*n*	5	6	10	6
Juveniles	Mean ± SE	11.76±1.17	5.34±1.27	4.02±1.82	4.50±1.92.
	range	9.50–13.45	1.39–9.57	0.69–12.83	0.9–9.28
	*n*	3	6	6	5

**Figure 3:**
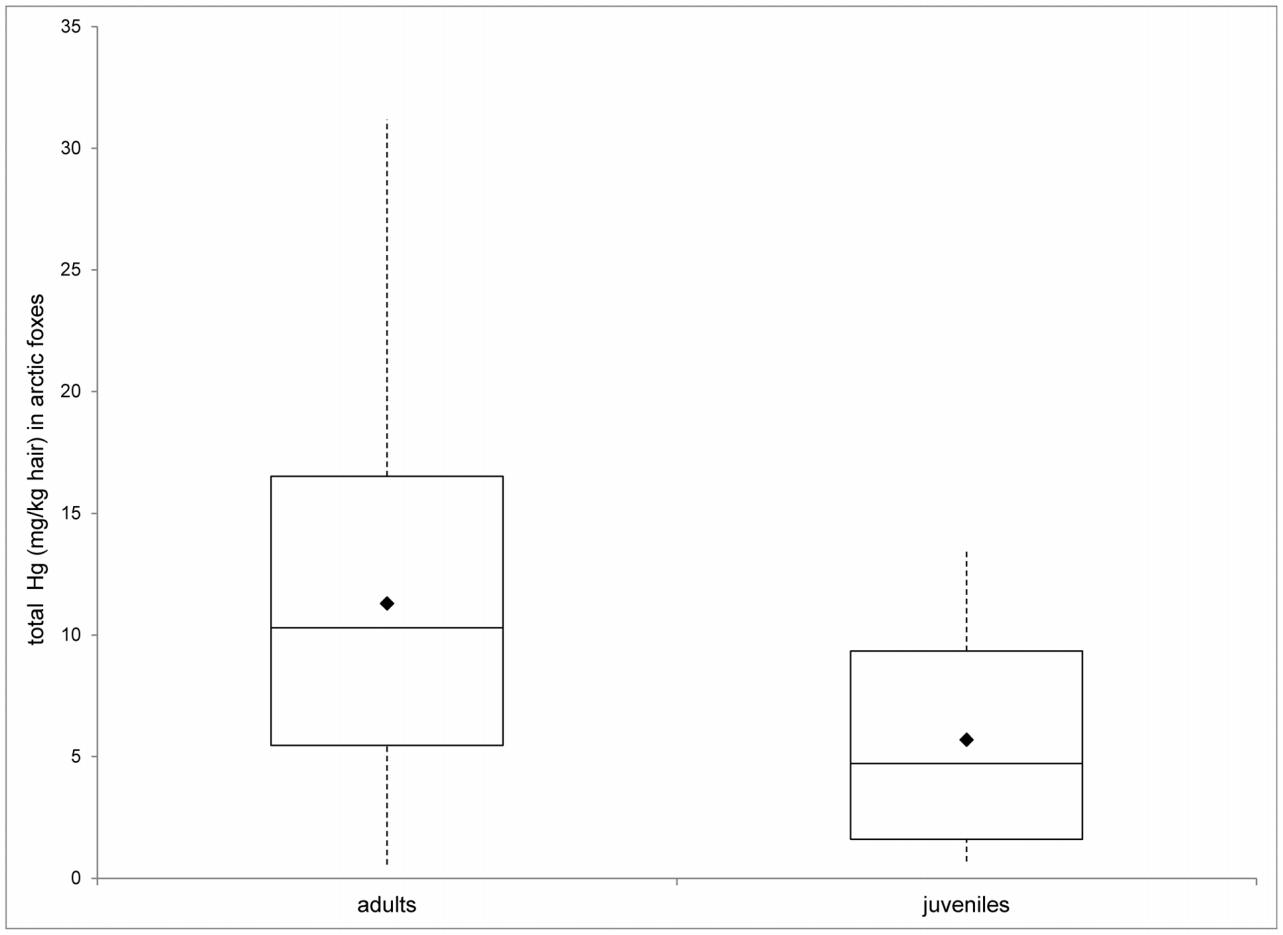
Differences in THg in hair (mg/kg d.w.) between adult and juvenile arctic foxes from the Commander Islands and Iceland. The data is displayed as in Figure 2.

THg concentrations increased with age (*F*_1,35_ = 9.21, *P* = 0.004), adult foxes having higher levels compared to juveniles (mean (SE) adults: 11.29 (1.62), juveniles: 5.70 (0.98)) (Figure 3). No sex differences or significant interaction between the sex and age groups and THg levels was observed.

**Figure 4:**
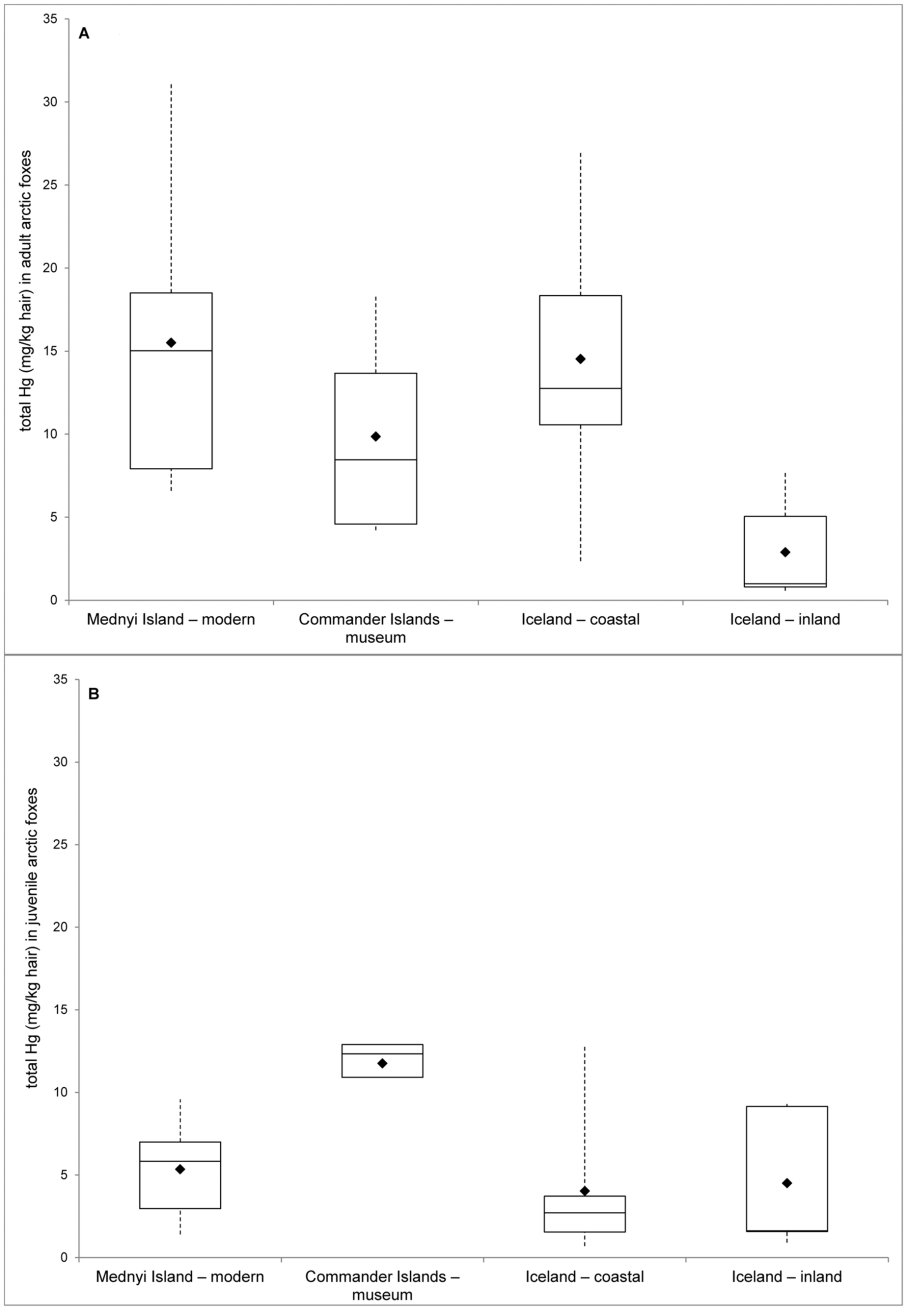
Age differences in levels of THg in hair (mg/kg d.w.) of arctic foxes originating from different ecotypes. Data for adult (A) and juvenile (B) foxes were analysed separately by ecotype. The data is displayed as in Figure 2.

When analysing the two age groups separately, no differences were found between adult males and females (mean (SE) adult males: 10.62 (2.34), adult females: 12.57 (2.64)). However, the significant differences between THg levels and ecotype (*F*_3,20_ = 5.83, *P* = 0.005, Figure 4A) persisted. Hair THg concentration did not differ between individuals with similar feeding strategies (marine food chain: Mednyi Island – modern, Iceland coastal and Commander Islands – museum; inland/continental food chain: Iceland inland and Commander Islands – museum) (Tukey-Kramer post-hoc test, all *P*>0.05). However, ecotype differences were significant (Tukey-Kramer post-hoc test, all *P*<0.05).

For juveniles, females and males had similar THg concentrations in their hair (Kruskal-Wallis test: χ^2^ = 2.08, *P* = 0.14; mean (SE) juvenile males: 3.81 (1.30), juvenile females: 5.55 (1.28)) and there was a slight difference between different populations with juveniles from Commander Islands – museum showing the highest THg concentration values (Table 2, Figure 4B). However, the trend did not reach statistical significance (Kruskal-Wallis test: χ^2^ = 6.40, *P* = 0.09).

### THg levels in prey species of Mednyi island foxes

The level of THg varied in relation to prey species and the tissues analysed (Table 3). Seal THg levels were variable but on average, high. Sample sizes were insufficient to compare museum and modern northern fur seal hair samples statistically, but no differences were observed. In the case of marine birds, the average Hg concentrations were lower than seals with the exception of two fulmars (Table 3).

**Table 3:** Level of total Hg (mg/kg d.w.) in the prey samples of the Mednyi island arctic foxes.

			total Hg ranges in mg/kg d.w. (*n*)			
Species (*n*)	sampling area	age (juvenile/adult/n.d.)	hair	dry muscle	dry placenta	dry liver
Northern fur seal(*Callorhinus ursinus*) (21)	Mednyi Island	1/3	5.08 (1)	1.02–2.38 (2)	1.19–1.68 (2)	
	Bering Island	13/2/2	0.64–10.35 (17)			
Pelagic cormorant(*Phalacrocorax pelagicus*) (1)	Mednyi Island	juvenile		0.79		
Northern fulmar(*Fulmarus glacialis*) (3)	Mednyi Island	all adults		0.10–2.87(2)		19.65–32.71 (2)
Glaucous-winged gull(*Larus glaucescens*) (2)	Mednyi Island	all adults		3.19–4.31 (2)		
Tufted puffin(*Lunda cirrata*) (1)	Mednyi Island	adult		1.41		
Pigeon guillemot(*Cepphus columba*) (1)	Mednyi Island	adult		1.14		
Fork-tailed storm petrel(*Oceanodroma furcata*) (2)	Mednyi Island	all adult		1.56–1.59 (2)		3.46 (1)
Sperm whale(*Physeter catodon*) (1)	Mednyi Island	juvenile		0.79		

n.d.: no data available.

## Discussion

In contrast to Mednyi Island, which from the fox point of view is a solely coastal environment with the only food source being marine life, Bering Island is insular but has both broader habitat and food choices available to the foxes. Iceland represents two ecotypes with inland foxes feeding primarily on small land mammals and birds and coastal foxes which have a similar diet to Mednyi Island foxes with the difference being that they can migrate to the inland ecotype. What makes Mednyi Island unique is that it represents the only arctic fox ecotype to have suffered declines, almost extinction, in recent history.

The Commander Islands were intensively disturbed as a hunting ground for northern fur seals and arctic foxes fur. Two villages were founded on the islands, one on the Bering Island (up to 800 inhabitants) and another, smaller one (up to 300 inhabitants), on the Mednyi Island, to house people who worked for the island fur farms. The farms were in operation on both islands from 1922 to 1962 [4]. Fox trapping and farming stopped on Mednyi Island at the end of 1960s, and the settlement was disbanded in 1972, while fox numbers were still high. The presence of humans and their commensals, particularly dogs, provide possible opportunities for pathogen introduction. However, no relevant pathogens that could explain the declines were observed and only few were detected in a low prevalence (*Toxoplasma gondii*). Thus, pathogens do not seem to be driving ecotype differences in population sustainability of arctic foxes.

Exposure to environmental pollutants such as Hg can have negative consequences for wildlife including accelerating local population extinctions [30]. The arctic foxes analyzed in this study represent four ecotypes: (1) the inland habitat type (Icelandic inland population) where foxes feed strictly on terrestrial vertebrates, (2) the mainland coastal ecotype (Iceland coastal population), where they feed on marine diet but can also switch to terrestrial sources by migrating inland, (3) the large island coastal ecotype (Commander Islands museum samples), where foxes depend on both marine and terrestrial mammals for food and (4) the purely coastal small island ecotype represented by the Mednyi Island population, completely reliant on a marine derived diet (marine birds, ocean invertebrate or mammals).

In general pollutant levels depend on factors such as age, sex, season, nutritional condition and feeding strategy. In the present study however, season, geographic origin and sex had no significant influence on the THg levels. The strongest correlation with high THg concentration was with a marine ecotype origin (10.47 mg/kg hair) versus inland (3.55 mg/kg hair). This suggests that the THg level differences reflect the foraging strategies and diet of arctic foxes (proportions of marine and terrestrial prey species) rather than variation in the overall THg burden of the foxes' environment which was also proposed for polar bears (*Ursus maritimus*) by [31] and may represent a general risk for top predators.

The ecotype profiles of THg exhibited broad similarity across geographical regions, suggesting that diet is a predictor of THg levels in this species. The results for THg are consistent with feeding ecology correlates with other persistent pollutants (e.g. PCBs) in Iceland [32] and Svalbard [33], where foxes living in coastal habitats had significantly higher contaminant levels. Even though mercury has always been a natural occurring element, temporal increases (e.g. through anthropogenic emissions or temperature related mercury releases through snow melt as consequence of climate change) could have an effect on exposed organisms due to a higher contamination risk caused by altered mercury fluxes in the Arctic [34]. The foxes of the Icelandic inland population sampled in this study are known to feed on uncontaminated sources like terrestrial birds and wood mice (*Apodemus sylvaticus*) and have no marine contribution to their diet [26] which could explain their low THg burden (3.55 mg/kg hair) and the small variation of THg values in contrast to the three coastal groups which varied strongly ranging from 0.7 to 31 mg/kg THg even though the mean THg was similar (about 10.47 mg/kg hair).

Of the four ecotypes, only the Mednyi Island foxes have restricted food choice and it is likely that their Hg levels are the most dependent on the fluctuations of Hg in the marine environment. Other studies on Hg levels in arctic biota including prey species of arctic foxes from Canada and Russia show that Hg concentrations depend heavily on time of sampling, age of individuals, duration and differences in local Hg exposure, species dependent degree of Hg accumulation and demeythylation capacity [22]. For example THg concentrations found in northern fur seals ranged from 3.00 to 61.2 mg/kg w.w. in liver, 0.40–1.57 mg/kg w.w. in kidney [35] and 2.9–7.6 µg/g d.w. in hair [36], between 1.3–21.2 mg/kg d.w. in hair of Steller sea lions (*Eumetopias jubatus*) [37], between 5.2–43.5 mg/kg d.w. in liver and 0.3–3.0 mg/kg d.w. in muscle of Northern fulmars (*Fulmarus glacialis*) [38]. The prey samples from Mednyi from this study were in these ranges, although the THg content of the museum seal hair samples had a wider range (0.64–10.35 mg/kg d.w.).

The Icelandic population is thriving and the Bering Island population is stable although Hg is present in all 3 ecotypes. The Mednyi arctic fox population is the only one that has experienced a steep decline recently though it currently remains low but stable. It is however remarkable, that after passing the population bottleneck, the diet of Mednyi arctic foxes changed considerably. Northern fulmars and storm petrels became the main food resource, the use of other bird species, marine invertebrates and otarid products having significantly decreased [7]. The Northern fulmar (*Fulmarus glacialis*) breeds on Siberian and Alaskan islands and winters widely throughout the Pacific, feeding mainly on invertebrates and some fish [39] whereas the two storm petrels species (*Oceanodroma furcata* and *O. leucorhoa*) are pelagic plankton eaters [40]. Due to their relatively lower position within the food chain it is very likely that these bird species are generally less contaminated with Hg or other pollutants than seals. However, northern fulmars have been reported to have higher contamination level than expected from their trophic levels [41], the highest Hg burden among arctic seabirds [42], [43]. Concentration of THg in tissues of fulmars can reach comparable level to seals (Table S5). Thus, reliance on these species exclusively as prey would expose Mednyi Island foxes to variable but chronically high THg levels as opposed to foxes consuming less contaminated prey or mixtures of contaminated or uncontaminated prey.

THg levels significantly increased with age in all ecotypes. It is well documented that Hg bioaccumulates in correlation with long life span of mammals due to longer time of exposure and also as consequence of low excretion rates [22]. Further, such age-related differences could also result from differences in the foraging behaviour between juvenile and adult arctic foxes. Since Hg bioaccumulates with animal age and biomagnifies within food webs, a relatively small increase in environmental Hg exposure over time could produce large and age-dependent increases in high trophic level species [44]. Furthermore out of all ecotype groups, the historical juvenile foxes from the Commander Islands showed the highest THg burden exceeding the adult concentrations from the same sampling period, which may explain observed skin abnormalities. Hair and skin accumulate methylmercury in which may serve as an excretory route in mammals. Many arctic foxes on the Mednyi Island have obviously bad fur quality (Figure 1B), rear dull hairs, desquamation of the skin, bald patches, in some cases foxes are almost completely bald. Similar changes were described in Iceland [45] where foxes with skin abnormalities are observed in coastal areas. Foxes on the Bering Island have similar skin abnormalities (Ploshnitsa *personal communication*). The correlation between hair THg concentrations and hair quality should be further investigated as it may influence winter survival rates.

Significant ecotype differences in Hg concentration were demonstrated among arctic foxes that are driven by feeding ecology as opposed to general environmental contamination. This may in part explain the decline of the Mednyi Island foxes. Thus, the diet choices available in each ecosystem determines Hg concentration in the arctic fox population and suggests conservation management strategies of foxes and other top predators should consider this aspect of animal health in developing conservation strategies for endangered carnivores.

## Supporting Information

Text S1 **Serological and molecular methods used on Mednyi Island arctic fox samples.**
(DOC)

Table S1 **Number of arctic fox individuals from different ecotypes sampled for THg measurements and used in this study.**
(DOC)

Table S2 **Prey species samples collected for THg measurements for this study.**
(DOC)

Table S3 **PCR conditions for the different pathogens screened.**
(DOC)

Table S4 **Primers used for pathogens detection and real time RT-PCR.**
(DOC)

Table S5 **Literature values for THg concentrations in tissues of Northern Fulmars and eared seals in North Pacific (mean ± SD,** ***n*** **). Values measured for dry weight are extra bold.**
(DOC)
