# Supplementary material for: Correlates between Feeding Ecology and Mercury Levels in Historical and Modern Arctic Foxes (Vulpes lagopus)
Source: PLoS One. 2013 May 6;8(5):e60879. doi: 10.1371/journal.pone.0060879 (PMC3645996; doi:10.1371/journal.pone.0060879)
Supplement: Text S1 — Serological and molecular methods used on Mednyi Island arctic fox samples. (DOC) [file pone.0060879.s001.doc]

**Serological methods used on Mednyi Island Arctic fox samples:**

***Toxoplasma gondii***

Enzyme linked immunosorbent assay (ELISA) was done with a commercially available kit for canine IgG for *Toxoplasma gondii* (Xema Co., LTD, Russia) according to manufacturer instruction. Two Western immunoblots using total antigen of *Toxoplasma gondii* or affinity purified surface antigens TgSAG1 were used as confirmatory tests following a previously published protocol [S1] using a serum dilution of 1:100 and an anti-dog IgG(H+L) peroxidase conjugate (Dianova, Hamburg, Germany).

***Neospora caninum***

Two Western immunoblots using total antigen of *Neospora caninum* or affinity purified surface antigens NcSRS2 were used following previously published protocols [S1,S2] using a serum dilution of 1:100 and an anti-dog IgG(H+L) peroxidase conjugate (Dianova, Hamburg, Germany).

**Canine Distemper Virus**

To detect antibodies against Canine Distemper Virus, direct neutralizing peroxidase-linked antibody (NPLA) assay was performed [S3]. The arctic foxes sera were diluted 1:100 anti-dog IgG(H+L)-Peroxidase from Dianova, Hamburg as a conjugate.

**Canine Parvovirus**

A haemagglutination assay (HA) was performed with virus strain UBI 265 p7 and pig erythrocytes. Pigs’ blood was collected in Alsever’s solution and stored at 4°C. Erythrocytes were washed with hemagglutination buffer (barbiturate-acetate, pH 6.2, with addition of bovine serum albumin 1 g/l and sodium azide 0.6 g/l). Test was performed in plastic 96-well plates. Serial 2-fold dilutions of virus were prepared in 25 µl of buffer, mixes with 25 µl of 0.5% erythrocyte suspension and incubated at room temperature for 45 min. The HA titer was expressed as the reciprocal of highest antigen dilution showing complete HA. Arctic foxes sera were diluted 5-folds with HA-buffer and incubated 1 h at 56°C, serial 2-fold dilution were made from these samples in 50 µl of antigen diluent, mixed with 25 µl of virus dilution and incubated 1h at room temperature. Than 50 µl of erythrocyte suspension was added, incubated at room temperature for 45 min, than readings were taken. Serum from a vaccinated dog was used as a positive control.

**PCR used on Mednyi Island Arctic fox samples**

**Usual PCR**

All cycling programs and the primers sequences are detailed in the Table S3 and S4 of the electronic supplementary material respectively. All samples were amplified using peqSTAR 96 Universal Gradient cycler (PEQLAB, Germany). Vaccine Epivax® SHPP+LT (ESSEX TEIRARZNEI, Germany) was used as a positive control for parvovirus.

**Reverse transcription and qPCR**

For the reverse transcription, depending on the sample type, 9 ng to 1.8 µg RNA was mixed with 200 U of M-MLV reverse transcriptase (Promega) and 20 pmol of random hexamer primers (Promega) in appropriate buffer containing 50mM Tris-HCl (pH 8.3), 7 mM MgCl2, 40 mM KCl, 10 mM DTT, 0.1 mg/ml BSA and 40 U of RNAse inhibitors, and 1 mM dNTPs (Fermentas). The reaction mix was incubated 1 hour at 37°C, reaction then was stopped by 5 min incubation at 95°C.

As samples of blood were not properly fixed for RNA preservation (blood clots fixed with 70% ethanol), we tested RNA eluate for the presence and amount of RNA by real time quantitative RT-PCR with primers for 18S ribosomal RNA. Specific primers for fox 18S RNA were used [S4]. We measured the amount of 18S RNA in two samples from free ranging Arctic foxes – one with the highest concentration of RNA in eluate and one with the lowest, and compared results with the amount of 18S RNA in properly fixed for preservation of RNA blood samples from farmed arctic foxes. Samples from the captive foxes (*n* = 4) were obtained during the slaughter on a fur farm, located in Moscow region. Blood was taken from jugular vein of adult foxes to PAXgene-tubes® (QIAGENE) and processed according to the manufacturer’s instructions. For the extraction of RNA PAXgene Blood RNA® kit (QIAGENE) was used.

A qPCR standard curve was generated using dilutions of 10^7^ to 10 copies of 18S ORF per µl obtained in usual PCR and cleaned with QIAquick PCR Purification kit (QIAGEN). 2 µl of pure reverse transcription product were added to 12.5 µl of Brilliant II SYBR® Green master mix 2x (Stratagene, USA), 0.4 µl of 10 mM forward and reverse primers, 0.375 µl of ROX reference dye (Invitrogen, USA) and molecular biology grade water up to 25 µl volume. All samples were analyzed on Mx3005P thermocycler (Agilent Technologies, USA). The cycling program is in Table S3. All samples and standard curves were analyzed in duplicate, control reactions with no reverse trancriptase added were performed for each sample.

As a positive control for Morbilliviruses we used RNA extracted from vaccine Epivax® SHPP+LT (Essex Tierarznei, Germany) and a positive sample from free ranging red fox (*Vulpes vulpes*) kindly provided by Veljko Nikolin. As a positive control for Caliciviruses we used a sample from hyena (*Crocuta crocuta*) kindly provided by Katja Goller.

**Supplementary references**

1. De Azevedo SS, De Jesus Pena HF, Alves CJ, De Melo Guimaraes Filho AA, Oliveira RM, et al. (2010) Prevalence of anti-*Toxoplasma gondii* and anti-*Neospora caninum* antibodies in swine from Northeastern Brazil. Rev Bras Parasitol Vet 19: 80–84.
2. Schares G, Wenzel U, Muller T, Conraths FJ (2001) Serological evidence for naturally occurring transmission of *Neospora canium* among foxes (*Vulpes vulpes*). Int J Parasit 31: 418–423. doi:10.1016/S0020-7519(01)00118-7.
3. Frölich K, Czupalla O, Haas L, Hentschke J, Dedek J, et al. (2000) Epizootiological investigations of canine distemper virus in free-ranging carnivores from Germany. Vet Microbiol 74: 283–292.
4. Rolland-Turner M, Farré G, Boué F (2006) Cloning of fox (*Vulpes vulpes*) Il2, Il6, Il10 and IFNgamma and analysis of their expression by quantitative RT-PCR in fox PBMC after in vitro stimulation by Concanavalin A. Vet Immunol Immunopathol 110: 369–375. doi:10.1016/j.vetimm.2005.10.006.
