# Supplementary material for: Correlates between Feeding Ecology and Mercury Levels in Historical and Modern Arctic Foxes (Vulpes lagopus)
Source: PLoS One. 2013 May 6;8(5):e60879. doi: 10.1371/journal.pone.0060879 (PMC3645996; doi:10.1371/journal.pone.0060879)
Supplement: Table S1 — Number of arctic fox individuals from different ecotypes sampled for THg measurements and used in this study. (DOC) [file pone.0060879.s002.doc]

| **Origin** | **Adults (*n*)** | **Juveniles (*n*)** | **Not determined age (*n*)** |
| --- | --- | --- | --- |
| Commander Islands – museum | 5 (1 ♀, 2 ♂, 2 n.d.) | 3 (3 n.d.) | 3 (3 ♀) |
| Mednyi Island – modern | 6 (3 ♀, 3 ♂) | 6 (3 ♀, 3 ♂) | - |
| Iceland – coastal | 10 (5 ♀, 5 ♂) | 6 (1 ♀, 5 ♂) | - |
| Iceland – inland | 6 (2 ♀, 4 ♂) | 5 (4 ♀,1 ♂) | 1 (1 ♂) |

adult: > 1 years; juvenile: <1 years; n.d.: no data available
