# Supplementary material for: Correlates between Feeding Ecology and Mercury Levels in Historical and Modern Arctic Foxes (Vulpes lagopus)
Source: PLoS One. 2013 May 6;8(5):e60879. doi: 10.1371/journal.pone.0060879 (PMC3645996; doi:10.1371/journal.pone.0060879)
Supplement: Table S2 — Prey species samples collected for THg measurements for this study. (DOC) [file pone.0060879.s003.doc]

| **Species (*n*)** | **Sampling area** | **Sampling year** | **Age (juvenile / adult / n.d.)** | **Tissue type** | | | |
| --- | --- | --- | --- | --- | --- | --- | --- |
|  |  |  |  | **hair** | **dry muscle** | **dry placenta** | **dry liver** |
| Northern fur seal  (*Callorhinus ursinus*) (21) | Mednyi Island | 2011 | 1 / 3 | 1 | 2 | 2 |  |
|  | Bering Island | 1935-1984 | 13 / 2 / 2 | 17 |  |  |  |
| Pelagic cormorant  (*Phalacrocorax pelagicus*) (1) | Mednyi Island | 2011 | juvenile |  | 1 |  |  |
| Northern fulmar  (*Fulmarus glacialis*) (3) | Mednyi Island | 2011 | all adults |  | 2 |  | 2 |
| Glaucous-winged gull  (*Larus glaucescens*) (2) | Mednyi Island | 2011 | all adults |  | 2 |  |  |
| Tufted puffin  (*Lunda cirrata*) (1) | Mednyi Island | 2011 | adult |  | 1 |  |  |
| Pigeon guillemot  (*Cepphus columba*) (1) | Mednyi Island | 2011 | adult |  | 1 |  |  |
| Fork-tailed storm petrel  (*Oceanodroma furcata*) (2) | Mednyi Island | 2011 | all adults |  | 2 |  | 1 |
| Sperm whale  (*Physeter catodon*) (1) | Mednyi Island | 2011 | juvenile |  | 1 |  |  |

n.d.: no data available
