# Supplementary material for: Correlates between Feeding Ecology and Mercury Levels in Historical and Modern Arctic Foxes (Vulpes lagopus)
Source: PLoS One. 2013 May 6;8(5):e60879. doi: 10.1371/journal.pone.0060879 (PMC3645996; doi:10.1371/journal.pone.0060879)
Supplement: Table S3 — PCR conditions for the different pathogens screened. (DOC) [file pone.0060879.s004.doc]

| **Number of samples** | **Sample type** | **Target** | **Method** | **Reaction volume (µl)** | **Primers conc. (mM)** | | **Enzyme (U)** | **dNTP (mM)** | **MgCl_2_ (mM)** | **Template (µl)** | **Cycling program** | | | | |
| --- | --- | --- | --- | --- | --- | --- | --- | --- | --- | --- | --- | --- | --- | --- | --- |
|  |  |  |  |  | **Fwd** | **Rev** |  |  |  |  |  |  |  |  |  |
|  |  |  |  |  |  |  |  |  |  |  | **Reverse transcription** | **Nr. cycles** | **Denaturation** | **Annealing** | **Elongation** |
| 13 adults | blood | *Brucella* spp. | PCR | 25 | 0,28 | 0,28 | 1,25 | 0,2 | 2,5 | 2 | - | 35 | 35s 95°C | 30s 50°C | 45s 72°C |
| 6 adults + 4 juveniles | feces | Canine Parvovirus | PCR | 25 | 0,4 | 0,4 | 1 | 0,4 | 3,5 | 5 | - | 10 | 30s 94°C | 30s 61°C | 30s 68°C |
|  |  |  |  |  |  |  |  |  |  |  |  | 25 | 30s 94°C | 30s 59°C | 30s 68°C |
| 13 adults + 5 juveniles | skin | Herpesvirus | Nested PCR | 25 | 0,4 | 0,4 | 1,25 | 0,2 | 1,5 | 1)4; 2)1 | - | 40 | 30s 94°C | 30s 60°C | 30s 72°C |
| 13 adults + 10 juveniles | blood | Morbilliviruses | One-step reverse transcription PCR | 20 | 0,5 | 0,5 | * | 0,2 | 1,6 | 3 | 30min 45°C | 35 | 15s 94°C | 30s 52°C | 30s 72°C |
| 13 adults + 10 juveniles | blood | Caliciviruses | One-step reverse transcription PCR | 20 | 0,5 | 0,5 | * | 0,2 | 1,85 | 3 | 30min 45°C | 35 | 15s 94°C | 30s 49°C | 30s 72°C |
| 13 adults + 10 juveniles | blood | Reoviruses | One-step reverse transcription PCR | 20 | 0,2 | 0,2 | * | 0,2 | 1,6 | 3 | 30min 45°C | 35 | 15s 94°C | 30s 50°C | 30s 72°C |
| 1 adult + 1 juvenile from  Mednyi Island;  4 adults from Russian fur farm | blood | 18S | Reverse transcription real time qPCR | 20 | 20 pmol random hexamer | | 200 | 1 | 7 | 9 | 1h 37°C | - | - | - | - |
|  |  |  |  | 25 | 0,16 | 0,16 | ** | ** | 2,5 | 4 | - | 40 | 30s 95°C | 30s 58°C | 30s 72°C |

*- 0.5 µl of SuperScript™ III RT/ Platinum® Tag mix (Invitrogen, USA)

**- 12.5 µl of 2x Brilliant II SYBR® Green qPCR Master Mix (Stratagene, USA)
