# Supplementary material for: Correlates between Feeding Ecology and Mercury Levels in Historical and Modern Arctic Foxes (Vulpes lagopus)
Source: PLoS One. 2013 May 6;8(5):e60879. doi: 10.1371/journal.pone.0060879 (PMC3645996; doi:10.1371/journal.pone.0060879)
Supplement: Table S4 — Primers used for pathogens detection and real time RT-PCR. (DOC) [file pone.0060879.s005.doc]

| **Target** | **Forward primer 5’ to 3’ sequence** | **Reverse primer 5’ to 3’ sequence** | **Tm** | **Amplicon length (pb)** | **Reference** |
| --- | --- | --- | --- | --- | --- |
| *Brucella* spp | ISP1: GGT TGT TAA AGG AGA GC | ISP2: GAC GAT AGC GTT TCA ACT TG | 50 | 650 | [S5] |
| Parvovirus | M1: GAA AAC GGA TGG GTG GAA AT | M2: AGT TGC CAA TCT CCT GGA TT | 59-61 | 201 |  |
| Herpesvirus | ILK: TCCTGGACAAGCAGCARNYSGCNMTNAA | IYG: CACAGAGTCCGTRTCNCCRTADAT | 60 | 268 | [S6] |
| Herpesvirus | TGV: TGTAACTCGGTGTAYGGNTTYACNGGNGT | IYG: CACAGAGTCCGTRTCNCCRTADAT | 60 | 233 | [S6] |
| Morbillivirus | ATCATGTTTATGATCACAGCGGT | ATTGGGTTGCACCACTTGTC | 52 | 429 | [S7] |
| Calicivirus | P290: GATTACTCCAAGTGGGACTCCAC | P289: TGACAATGTAATCATCACCATA | 49 | 318 | [S8] |
| Reovirus | S2-26: GCG CTG CGT TCC TAT TCAAGA CT | S2-563: TAG TTC ATG AGA AGC TGGTTC AC | 50 | 538 | [S9] |
| 18S | TTCAAGAACGAAAGTCGGAG | TGAGTCAAATTAAGCCGCAG | 58 | 227 | [S4] |

**Supplementary references**

1. Rolland-Turner M, Farré G, Boué F (2006) Cloning of fox (*Vulpes vulpes*) Il2, Il6, Il10 and IFNgamma and analysis of their expression by quantitative RT-PCR in fox PBMC after in vitro stimulation by Concanavalin A. Vet Immunol Immunopathol 110: 369–375. doi:10.1016/j.vetimm.2005.10.006.
2. Antiabong JF, Yakubu B, Owolodun OA, Bertu W, Ocholi RA (2009) Molecular detection of *Brucella* spp. from broth culture of clinical samples in Nigeria: Its role in vaccine quality control. African J Biotechnol 8: 5661–5665.
3. VanDevanter DR, Warrener P, Bennett L, Schultz ER, Coulter S, et al. (1996) Detection and analysis of diverse herpesviral species by consensus primer PCR. J Clin Microbiol 34: 1666–1671.
4. Barrett T, Visser IK, Mamaev L, Goatley L, Van Bressem MF, et al. (1993) Dolphin and porpoise morbilliviruses are genetically distinct from phocine distemper virus. Virology 193: 1010–1012.
5. Jiang X, Huang PW, Zhong WM, Farkas T, Cubitt DW, et al. (1999) Design and evaluation of a primer pair that detects both Norwalk- and Sapporo-like caliciviruses by RT-PCR. J Virol Methods 83: 145–154.
6. Muscillo M, La Rosa G, Marianelli C, Zaniratti S, Capobianchi MR, et al. (2001) A new RT-PCR method for the identification of reoviruses in seawater samples. Water Res 35: 548–556.
