# Supplementary material for: Correlates between Feeding Ecology and Mercury Levels in Historical and Modern Arctic Foxes (Vulpes lagopus)
Source: PLoS One. 2013 May 6;8(5):e60879. doi: 10.1371/journal.pone.0060879 (PMC3645996; doi:10.1371/journal.pone.0060879)
Supplement: Table S5 — Literature values for THg concentrations in tissues of Northern Fulmars and eared seals in North Pacific (mean ± SD, n ). Values measured for dry weight are extra bold. (DOC) [file pone.0060879.s006.doc]

| **Species** | **Reference** | **Place** | **Tissue** | | | |
| --- | --- | --- | --- | --- | --- | --- |
|  |  |  | **liver** | **kidney** | **muscle** | **feathers/hair** |
| Northern Fulmar (*Fulmarus glacialis*) | [S10] | Canadian Arctic, Cape Vera and Prince Leopold Island | **11.6 ± 0.77 (8) µg/g dw** |  |  |  |
| Northern Fulmar (*Fulmarus glacialis*) | [S11] | Baffin bay, Canada | 3.41 ± 1.6 (10) µg/g ww |  | 0.39 ± 0.14 (10) µg/g ww |  |
| Northern Fulmar (*Fulmarus glacialis*) | [S12] | Norway | 3.0 ± 2.7 (15) µg/g ww |  |  |  |
| Northern Fulmar (*Fulmarus glacialis*) | [S13] | Swalbard, Norway | 2.57 ± 0.17 (10) µg/g ww |  |  |  |
| Northern Fulmar (*Fulmarus glacialis*) | [S14] | Aleutian Islands | **24.6 ± 10 (3) µg/g dw** |  |  |  |
| Northern Fulmar (*Fulmarus glacialis*) | [S15] | North Pacific | **14.2 ± 10 (15) µg/g dw** | **6.7 ± 3 (5) µg/g dw** | **1.4 ± 1 (5) µg/g dw** | **4.8 ± 2.4 (17) µg/g dw** |
| Northern Fulmar (*Fulmarus glacialis*) | Our study | Mednyi Island | **19.65** – **32.71 (2) µg/g dw** |  | **0.1** – **2.87 (2) µg/g dw** |  |
| Northern fur seal (*Callorhinus ursinus*) | [S16] | Sanricu, Japan | 35.4 ± 30.4 (50) µg/g ww | 1.15 ± 0.42 (50) µg/g ww | 0.51 ± 0.22 (50) µg/g ww |  |
| Northern fur seal (*Callorhinus ursinus*) |  | Pribilof Island, Alaska | 26.7 ± 18.1 (17) µg/g ww | 0.98 ± 0.33 (17) µg/g ww | 0.36 ± 0.09 (17) µg/g ww |  |
| Northern fur seal (*Callorhinus ursinus*) | [S17] | Sanricu, Japan | **165 ± 132 (24) µg/g dw** | **4.4 ± 1.4 (20) µg/g dw** | **1.7 ± 0.5 (20) µg/g dw** | **4.9 ± 1.1 (20) µg/g dw** |
| Northern fur seal (*Callorhinus ursinus*) | Our study | Commander Islands |  |  | **1.02** – **2.38 (2) µg/g dw** | **0.64** – **10.35 (18) µg/g dw** |
| Steller sea lion (*Eumetopias jubatus*) | [S18] | Aleutian Islands |  |  |  | **1.78 ± 0.8 (16) µg/g dw** |

**Supplementary references**

1. Braune BM, Mallory ML, Gilchrist HG, Letcher RJ, Drouillard KG (2007) Levels and trends of organochlorines and brominated flame retardants in Ivory gull eggs from the Canadian Arctic, 1976 to 2004. Sci Total Environ 378: 403–417. doi:10.1016/j.scitotenv.2007.03.003.
2. Campbell LM, Norstrom RJ, Hobson KA, Muir DCG, Backus S, et al. (2005) Mercury and other trace elements in a pelagic Arctic marine food web (Northwater Polynya, Baffin Bay). Sci Total Environ 351: 247–263. doi:10.1016/j.scitotenv.2005.02.043.
3. Knudsen LB, Borga K, Jorgensen EH, Van Bavel B, Schlabach M, et al. (2007) Halogenated organic contaminants and mercury in northern fulmars (*Fulmarus glacialis*): levels, relationships to dietary descriptors and blood to liver comparison. Environ Pollut 146: 25–33. doi:10.1016/j.envpol.2006.06.017.
4. Jaeger A, Blanchard P, Richard P, Cherel Y (2009) Using carbon and nitrogen isotopic values of body feathers to infer inter- and intra-individual variations of seabird feeding ecology during moult. Mar Biol 156: 1233–1240. doi:10.1007/s00227-009-1165-6.
5. Ricca MA, Miles AK, Anthony RG (2008) Sources of organochlorine contaminants and mercury in seabirds from the Aleutian archipelago of Alaska: inferences from spatial and trophic variation. Sci Total Environ 406: 308–323. doi:10.1016/j.scitotenv.2008.06.030.
6. Kim EY, Murakami T, Saeki K, Tatsukawa R (1996) Mercury levels and its chemical form in tissues and organs of seabirds. Arch Environ Contam Toxicol 30: 259–266.
7. Noda K, Ichihashi H, Loughlin TR, Baba N, Kiyota M, et al. (1995) Distribution of heavy metals in muscle, liver and kidney of northern fur seal (*Callorhinus ursinus*) caught off Sanriku, Japan and from the Pribilof Islands, Alaska. Environ Pollut 90: 51–59.
8. Ikemoto T, Kunito T, Watanabe I, Yasunaga G, Baba N, et al. (2004) Comparison of trace element accumulation in Baikal seals (*Pusa sibirica*), Caspian seals (*Pusa caspica*) and northern fur seals (*Callorhinus ursinus*). Environ Pollut 127: 83–97.
9. Castellini JM, Rea LD, Lieske CL, Beckmen KB, Fadely BS, et al. (2012) Mercury concentrations in hair from neonatal and juvenile steller sea lions (*Eumetopias jubatus*): implications based on age and region in this Northern Pacific marine sentinel piscivore. EcoHealth 9: 267–277. doi:10.1007/s10393-012-0784-4
